# The Association Between the Subjective Exercise Experience of Chinese Women Participating in Square Dance and Group Cohesion: The Mediating Effect of Income

**DOI:** 10.3389/fpsyg.2021.700408

**Published:** 2021-10-12

**Authors:** Yuting Sun, Peiyao Ji, Yan Wang, Hongying Fan

**Affiliations:** ^1^School of Art, Beijing Sport University, Beijing, China; ^2^School of Psychology, Beijing Sport University, Beijing, China

**Keywords:** Chinese square dance, middle-aged women, subjective exercise experience, group cohesion, subjective well-being

## Abstract

**Background:** Chinese square dance has become well known worldwide in recent years, and most participants are women who dance with a group in their communities. In particular, middle-aged women may have physical and mental health problems, and participating in square dance may increase women’s positive subjective well-being and decrease their negative emotions, which may improve their health over the long term. In addition, participating in square dance can promote group cohesion. Our study aimed to examine the relationship between the subjective exercise experience of participating in square dance and group cohesion and whether some variables (e.g., age, education, duration, income level, and work) play a role as mediators in the association with subjective exercise experience and group cohesion.

**Methods:** In total, 1,468 Chinese women from 31 provinces and 82 cities participated in this study by completing an online questionnaire. The questionnaire consisted of a subjective exercise experience questionnaire and a group environment questionnaire. We analyzed the collected data and built a statistical model.

**Results:** (a) Square dance satisfied women’s physical and psychological needs partly; (b) positive well-being (PWB) was positively correlated with group cohesion, and fatigue was negatively correlated with group cohesion; and (c) the income level was a partial mediator of the relationship between group cohesion and subjective exercise experience.

**Conclusion:** Chinese women have different motivations for participating in square dance. Because this activity can help meet women’s physical and psychological needs, an increasing number of individuals worldwide participate in square dance. As women’s subjective well-being increases, group cohesion increases, and vice versa. Moreover, the subjective exercise experience remains a significant predictor of group cohesion after including income level as a mediator, suggesting that the model indicates partial mediation.

## Introduction

### Chinese Square Dance

Chinese square dance is a fitness activity with distinctive traditional Chinese characteristics that spontaneously occurs *via* grassroot efforts and has been widely acknowledged by people since 1978. By the time Beijing held the Olympics Games in 2008, Chinese square dance flourished in communities in almost all parts of China.

Square dance is defined as an aerobic group activity with music and various dance types that improves physical health and mental health, and the suitable duration of this exercise ranges from 20min to an hour (General Administration of Sport of China, 2020). Square dance is a form of amateur dance performed popularly among Chinese middle-aged and elderly women in public places (Xie et al., 2021). Thus far, Chinese square dance has gradually spread worldwide.

Given the charm of square dance, a unique form of exercise, fitness campaigns have achieved sustainable development. In China, there are different levels of square dance competitions, such as “Dancing China” leagues and competitions, in different categories of adults, including disabled people since 2017 (General Administration of Sport of China, 2020). Relatively formal and consistent square dance group competitions are held in both urban and rural regions of China. According to one China Central Television program, there were over 100 million fans of square dance (Hu, 2013). More than 100 million people – dubbed “dancing aunties” because they are primarily older women – took over squares and park to tango, waltz, and grind from flamenco to traditional Chinese dance every day (Kuwait Times, 2018). However, the Chinese show Chinese square dance in either the Louvre in France or the Red Square in Moscow, which could be identified as a new image of China worldwide (English China News, 2014).

One of the central United Nations Sustainable Development Goals is gender equality for females as being a woman is a risk factor for physical and psychological health problems, social issues, etc., particularly in developing countries (United Nations, 2015). In recent years, studies worldwide have indicated that women are at an increased risk of developing a wide variety of psychiatric disorders and psychological health problems (Collier et al., 2020). Women, especially middle-aged and elderly women, have psychological problems, such as depression, anxiety, and psychological fatigue (PF; Choi et al., 2015; Kim and Park, 2018; Kalhan et al., 2020). The U.S. Federal Interagency Forum on Aging-Related Statistics (2010) found that almost 18% of older women presented clinical symptoms of depression in 2006.

Compared to males, females always report fatigue in subjective exercise experiences caused by exercise (Ivancic et al., 2016), and elderly women are more prone to fatigue than men (Hardy and Studenski, 2010). Moreover, evidence suggests that the prevalence of fatigue among women aged over 45years was 33.9% in China (Jing et al., 2015) and that middle-aged Chinese women have physical and mental exhaustion symptoms in their daily life (Kong et al., 2019). Although young women’s, middle-aged women’s, and elderly women’s psychological problems may be related to work stress, menopausal symptoms, etc. (Melchior et al., 2007; Howard et al., 2014), attending physical and group activities, such as square dance, could significantly enhance the happiness index of women and effectively improve their psychological problems (Zhang et al., 2014). In addition, other studies found that moderate-intensity square dance exercise has effects on cognitive function and quality of life in older Chinese women with mild cognitive impairment, indicating that square dance can increase the quality of life due to a relationship between square dance exercise and cognition (Song et al., 2018; Chang et al., 2021).

### Motivation

Chinese square dance has become one of the most popular exercise formats in China. People participate in this physical and group activity for different reasons, including fitness, socialization, and self-satisfaction.

The motivation to engage in physical activity is often related to physical and psychological health, weight management, and fitness (Allender et al., 2006), and group-based settings are valuable in promoting physical activity when individuals perceive high groups or when they prefer to exercise in a true group (Spink et al., 2010). Notably, young women engage in physical activity due to their body image and motivation for weight loss (O’Dougherty et al., 2010), while middle-aged women focus on physical fitness and activity (Sit et al., 2008). In addition, female exercisers aged 65–96 living in rural areas had significantly higher motivation related to fitness, health, etc. (Janssen et al., 2014).

Second, spending time with friends and making new friends also served as motivation for participating in square dance (Cong, 2019). Dance exercise, such as square dance, offers women the opportunity to participate in a group, which enables them to experience social relationships, thereby lowering the risk of psychological diseases and improving subjective well-being (Paulson, 2005). Moreover, the association between physical activity and social practices can encourage people to be physically active and provide better results in adherence exercise programs in the general population (Rosa et al., 2015).

Finally, participating in social group activities and being accepted by a social group increase self-worth (Baumeister and Vohs, 2002; Stavrova and Luhmann, 2016). For example, working women reported an improved self-worth even though they attended a web-based group activity (Mailey et al., 2016).

### Subjective Exercise Experience

The subjective exercise experience is understood as follows: The stimulus properties of the exercise environment may give rise to subjective interpretations of physical symptoms during and following physical activity, and these stimuli produce subjective experiences that vary along a dimension other than positive and negative dimensions (McAuley and Courneya, 1994).

Psychological or subjective well-being has been defined as people’s overall evaluations of their lives and their emotional experiences (Diener et al., 2017) and is a multidimensional concept that refers to people’s evaluative judgments of their quality of life, “life satisfaction,” and emotional or affective states, such as happiness (Dolan and White, 2007). On the one hand, the positive effects of physical exercise on psychological well-being have been proven in previous research (Penedo and Dahn, 2005), and most research focused on physical exercise that may result in a positive effect, especially well-being (Gauvin and Rejeski, 1993; McAuley et al., 1996; Fox, 2000; Focht and Hausenblas, 2001; Thomas and Davies, 2007; Reed and Buck, 2009). Meanwhile, participating in a group activity could also enhance women’s psychological health and subjective well-being (Okun and George, 1984; Pinquart and Sörensen, 2000; Glass et al., 2006; Dai et al., 2013; Zhang and Zhang, 2015). For example, women in an aerobic exercise group showed higher positive well-being (PWB) than non-exercisers in a control group (Arbinaga et al., 2018). Similarly, older women exhibit significant growth in PWB after performing physical group activities over a 6-month period (McAuley et al., 2005). Therefore, square dance is a physical group activity that increases women’s positive effect and is gradually becoming a unique way for women to engage in entertainment and exercise.

In addition, fatigue is defined as individuals’ perceptions of somatic states (fatigue, pain) that can also be perceived as subjective feeling states (Clore et al., 1987; Gauvin and Brawley, 1993). Previous studies have demonstrated that physical group activities can help women decrease negative psychological effect (Domene et al., 2016). For example, middle-aged women with climacteric symptoms and women with breast cancer reported decreased PF when they participated in physical group activities (Canário et al., 2012; Boing et al., 2018). In addition, a recent study showed that performing square dance decreased negative effect, such as depression, among perimenopausal women (Gao et al., 2015). However, whether square dance improves women’s PF and the relationship between square dance and PF remain unclear.

### Group Cohesion

Group cohesion, which is a fundamental need of humans, implies that people need to belong (Baumeister and Leary, 1995). Previous studies defined group cohesion in many ways (Gross and Martin, 1952; Mullen and Copper, 1994; Mikulincer and Shaver, 2007). In this study, the concept of group cohesion is considered a dynamic process reflected in the shared pursuit of common objectives to satisfy members’ needs and place people together (Carron and Hausenblas, 1998) because it is consistently used in physical activity promotion and research.

A close relationship exists between physical group activities and group cohesion (May et al., 2008; Caperchione et al., 2011), and Xie et al. (2021) found that women participating in square dance exhibited enhanced group belonging, which strengthens this interpretation. In addition, square dance is a group activity spontaneously organized by Chinese women in their daily lives. The attraction of social and task cohesion may encourage middle-aged women to perform physical activity (Smith-Ray et al., 2012). When participants set group goals, the group cohesion and positive effects of physical activity are facilitated (Burke et al., 2006; Estabrooks et al., 2012), and friendly competition in a group activity may further promote minority women’s perceptions of group cohesion (Harden et al., 2014).

### The Relationship Between the Subjective Exercise Experience and Group Cohesion

Although few previous studies have investigated the relationship between subjective exercise experience and group cohesion, the relationship between positive and negative subjective exercise experience and group cohesion has been shown.

First, positive psychological effects, such as PWB, are positively correlated with group cohesion, which has been clearly demonstrated in previous studies. For example, Delhey and Dragolov (2016) found that Europeans in more cohesive societies are happier and psychologically healthier, which increases the capacity of their citizenry to create togetherness and solidarity among their members, thereby improving social cohesion. Second, women participating in square dance gain higher group belonging in square dance teams, enabling them to achieve a higher level of subjective well-being (Xie et al., 2021) because women who participate in social organizations and play active roles can enjoy higher levels of subjective well-being (Putnam and Harvey, 2000).

On the other hand, relatively limited research investigated the relationship between negative psychological effect and group cohesion among women, and the direct relationship between fatigue and group cohesion has not been proven in previous studies.

In addition, different variables may affect the results of studies. For instance, Bahou (2017) found that education is considered a vital vehicle for promoting positive well-being and social cohesion. Second, most studies recognized that a higher duration of performing physical activity was related to higher group cohesion (Steca et al., 2013). Therefore, our research considers not only the duration of belonging to a square dance team but also more variables (e.g., age, education, duration, income level, and work) during the data collection state to identify the variables that may influence the relationship between the subjective exercise experience and group cohesion among square dance teams. We hypothesized that (1) the motivation for participating in a square dance group could be explained by the benefits on physical and psychological health partly; (2) the positive aspect of the subjective exercise experience is positively related to group cohesion, whereas the negative aspect of the subjective exercise experience is negatively related to group cohesion (Kim et al., 2020); and (3) some variables (e.g., age, education, duration, income level, and work) play a role as mediators in the association between the subjective exercise experience and group cohesion. The Subjective Exercise Experience Scale score was regarded as the predictor (*PWB and PF*), while the Group Environment Questionnaire score was regarded as the dependent variable [*group integration-task (GI-T), group integration-social (GI-S), individual attractions to group-tasks (ATG-T), and individual attractions to group-social (ATG-S)*]. To test the above hypotheses, we performed the following analysis.

## Materials and Methods

### Participants

This study strictly adhered to the operation rules of standard biosecurity and institutional safety. Before the formal questionnaire, we obtained informed consent from the participants. Through online recruitment, 1,468 female participants were invited from 31 provinces of China (e.g., Northeast: Liaoning et al.; East: Beijing et al.; West: Qinghai et al.; and Center: Hubei et al.) and abroad from October 1 to October 24, 2019. To explore the effect of various demographic characteristics on the association between the subjective exercise experience and group cohesion, several choice (e.g., How much money could you earn every month? What is your highest educational level?) and fill-in (e.g., How old are you? What is your job? What have you gained from participating in a square dance group, and When did you begin to participate in a square dance group?) questions were designed for all participants.

The validity of the data was considered; thus, 77 samples were deleted based on time (>120s) and age (≥18years) limitations. In addition, for the purpose of this study, questionnaires with self-reported answers noting that the participant did not participate in a square dance group or responses with missing information were also regarded as invalid (114 samples). Although sampling was not limited to females, only a fraction of the data (102 samples) was submitted by males. Therefore, these responses were not included in the analysis. After eliminating incomplete data, we finally included 1,166 participants. Based on the income level (e.g., less than 2,000 yuan, 2,001–3,500 yuan, 3,501–5,000 yuan, 5,001–6,500 yuan, 6,501–8,000 yuan, and more than 8,000 yuan), the age of the participants can be described as 50.02±10.25, 58.26±8.19, 59.80±8.82, 56.54±11.77, 54.56±10.65, and 53.50±8.07, respectively. The length of the participation time can be described as 5.17±5.76, 5.76±5.86, 5.19±5.53, 5.03±4.74, 3.87±4.33, and 3.00±3.88. By virtue of policy indicating that women’s retirement age is 55years, 566 participants were retired (Interim Measures of the State Council on the Placement of Old, Weak, Sick and Disabled Cadres, 1978; Interim Measures of the State Council on Workers’ Retirement and Resignation, Development, 1978), and the item related to work did not apply (Table 1).

**Table 1:** Demographic information of the participants (*N*=1,166).

Demographic measures		Income (*yuan*)					
		≤2,000	2,001–3,500	3,501–5,000	5,001–6,500	6,501–8,000	>8,000
Education (*n*)	Primary	8	1	0	0	0	1
	Junior high	111	117	33	3	2	4
	Senior high	80	216	139	25	3	9
	Undergraduate	18	67	127	112	33	33
	Higher than undergraduate	2	2	3	5	1	11
Location (*n*)	Northeast	2	4	1	2	2	2
	East	70	300	263	121	26	44
	West	33	27	11	9	1	9
	Center	103	57	20	6	8	2
	Abroad	11	15	7	7	2	1
Work (*n*)	Government	0	0	8	4	3	5
	Government-sponsored institution	6	14	44	48	7	12
	Retiree	36	238	182	61	18	23
	Enterprise	14	80	46	18	3	10
	Individually owned business	7	11	3	1	5	7
	Military	0	0	0	0	0	0
	Framer	70	17	2	0	0	0
	Student	6	1	0	0	0	0
	Full-time house work	80	42	17	13	3	1
Age (*M±SD*)		50.02±10.25	58.26±8.19	59.80±8.82	56.54±11.77	54.56±10.65	53.50±8.07

### Questionnaire

#### Subjective Exercise Experience

The subjective exercise experience was measured using the Subjective Exercise Experience Scale (SEES; McAuley and Courneya, 1994), which was highly reliable (*α*_PWB_=0.86, *α*_PF_=0.88) in college students (age *M*=20.78years, *SD*=2.18). The factory, development, and preliminary validation of two factors, namely PWB and PF, has been shown using data collected from middle-aged (*M*=55years) exercisers. In this study, the subjective exercise experience was measured using a subset of 8 items measuring two dimensions of the SEES, and each dimension contained four items. Additionally, the Chinese version of the SEES has been repeatedly confirmed to have good reliability (average *α*=0.86) and validity (Zhou and Xie, 2016). These two dimensions were scored on a seven-point Likert scale ranging from *not at all* (1) to *very much so* (7).

#### Group Cohesion

Group cohesion was measured using the Group Environment Questionnaire 15 items based on the conceptual model, which contains the following four constructs: *GI-T, GI-S, ATG-T, and* ATG-S (Carron et al., 1985). Its validity is considered good (*ɑ*_ATG-T_=0.65, *ɑ*_ATG-S_=0.64, *ɑ*_GI-T_=0.71, and *ɑ*_GI-S_=0.72), and its reliability was tested among athletes. The original GEQ was adjusted to Chinese by Hongyu (2008), and this version is scored on a 7-point Likert scale to indicate the extent of agreement with each item from extremely agree (7) to extremely disagree (1). The Chinese version of the GEQ also has excellent internal consistency (*ɑ*_ATG-T_=0.76, *ɑ*_ATG-S_=0.75, *ɑ*_GI-T_=0.85, and *ɑ*_GI-S_=0.78), test–retest reliability (*r*_ATG-T_=0.70, *r*_ATG-S_=0.80, *r*_GI-T_=0.74, and *r*_GI-S_=0.70), and construct and criterion validity.

### Statistical Analysis

Open question item 22, i.e., “What did you gain from participating in a square dance group?,” was analyzed through three steps referred to as objective grounded theory (OCT, Strauss and Corbin, 1998), including open coding, axial coding, and selective coding in sequence. Group cohesion (GI-S, ATG-S, GI-T, and ATG-T) and the subjective exercise experience (PWB and PF) across the groups, which were divided by demographics (e.g., age, education, and income level) and duration, were analyzed using a MANOVA with IBM SPSS version 24.0 (IBM Corp., 2017).

According to Weston and Gore (2006), a major advantage of structural equation modeling (SEM) over a general linear model is that SEM considers error variables. Specifically, SEM allows researchers to model both variability common to a latent variable (i.e., error-free scores) and variability not explained by a latent variable (i.e., error). Moreover, SEM enables the creation of weighted aggregate variables of targeted constructs (Hawes et al., 2019). Therefore, we conducted and analyzed this single mediation model using Amos version 23.0 (IBM SPSS, 2014). The analyses were carried out using the recommended two-step approach to SEM. The first step involved testing the measurement model using confirmatory factor analyses (CFA). The purpose of the measurement model is to test and observe the relations between the manifest variables and the relations between these variables and the hypothesized latent variables. The second step involved analyses of the full structural equation models. The purpose of this step is to test the hypothesized interrelations between factors that are similar in some ways to general linear regression models (Kline, 2015). Specifically, the default maximum likelihood estimation (MLE) procedures were considered. All analyses were conducted using raw (continuous) scores. A modification index was required for Chi-squared values ≥10. We used the following three goodness-of-fit statistics to compare our CFA models and determine the model fit: (1) root-mean-square error of approximation (RMSEA), (2) comparative fit index (CFI), and (3) standardized root mean residual (SRMR). Notably, we also report the Chi-squared values (53.911) and degrees of freedom values (11) for comparison purposes, but due to the large sample size (>200), we did not interpret the statistically significant results in any meaningful way (Kline, 2015).

To test the mediating role of the income level in the association between the subjective exercise experience and group cohesion, the pathways of this model should be tested separately following the method outlined by Preacher and Hayes (2008). The bootstrapping technique was suggested by Hayes (2013) for hypothesis testing and effect size estimation. The indirect effect is considered significant when the CI (95%) does not contain the number zero (Hayes, 2013). Alpha was set to 0.05 for all analysis. Power analyses were conducted to determine the minimum sample size needed to detect a medium effect size with *α*=0.05 and *p*=0.95 (Soper, 2018).

## Results

Based on the analytic framework of the OGT, the responses to item 22 can be summarized as a safety need (fitness and health), attachment need (social circle), esteem need (sense of identity), cognition need (knowledge acquisition), aesthetics need (artistic accomplishment), and self-actualization need (sense of honor and patriotic sentiment) based on Maslow’s hierarchy of needs (Maslow, 1958), and PWB was added as the seventh item (see Table 2).

**Table 2:** Answers to question 22 measuring motivation participating in square dance between different income levels.

Hierarchy of needs (*n*)		Income (yuan)					
		≤2,000	2,001–3,500	3,501–5,000	5,001–6,500	6,501–8,000	>8,000
Safety	e.g., I strengthened my body.	124	265	208	95	20	37
Attachment	e.g., I made more friends.	40	101	75	40	7	12
Esteem	e.g., I became more confident with my performance.	6	8	4	4	2	0
Cognition	e.g., I gradually improved my dance skills.	17	37	30	19	5	12
Aesthetics	e.g., I cultivated my artistic aesthetics.	11	23	23	8	2	2
Self-actualization	e.g., I took pride in my or our teams achievement.	3	2	0	1	0	0
Positive well-being	e.g., It makes me feel happy.	123	238	157	68	23	34

Table 3 shows the bivariate zero-order correlations between all variables. Only the income level and education were significantly correlated with either group cohesion or the subjective exercise experience. The correlation between the income level and location was significantly weak (rs=0.332, *p*<0.001).

**Table 3:** Pearson correlations among the demographic, group cohesion, and subjective exercise experience variables.

Variables	1	2	3	4	5	6
1. Age	1.000					
2. Duration of exercise	0.206^**^	1.000				
3. Education	−0.048	−0.124^**^	1.000			
4. Income level	0.067^*^	−0.071^**^	0.527^**^	1.000		
5. Group cohesion	0.012	0.084^**^	−0.080^**^	−0.106^**^	1.000	
6. Subjective exercise experience	−0.116^**^	0.048	−0.106^**^	−0.144^**^	0.113^**^	1.000

All values represent standardized estimates.

** *p*<0.01;

* *p*<0.05.

**Figure 1:**
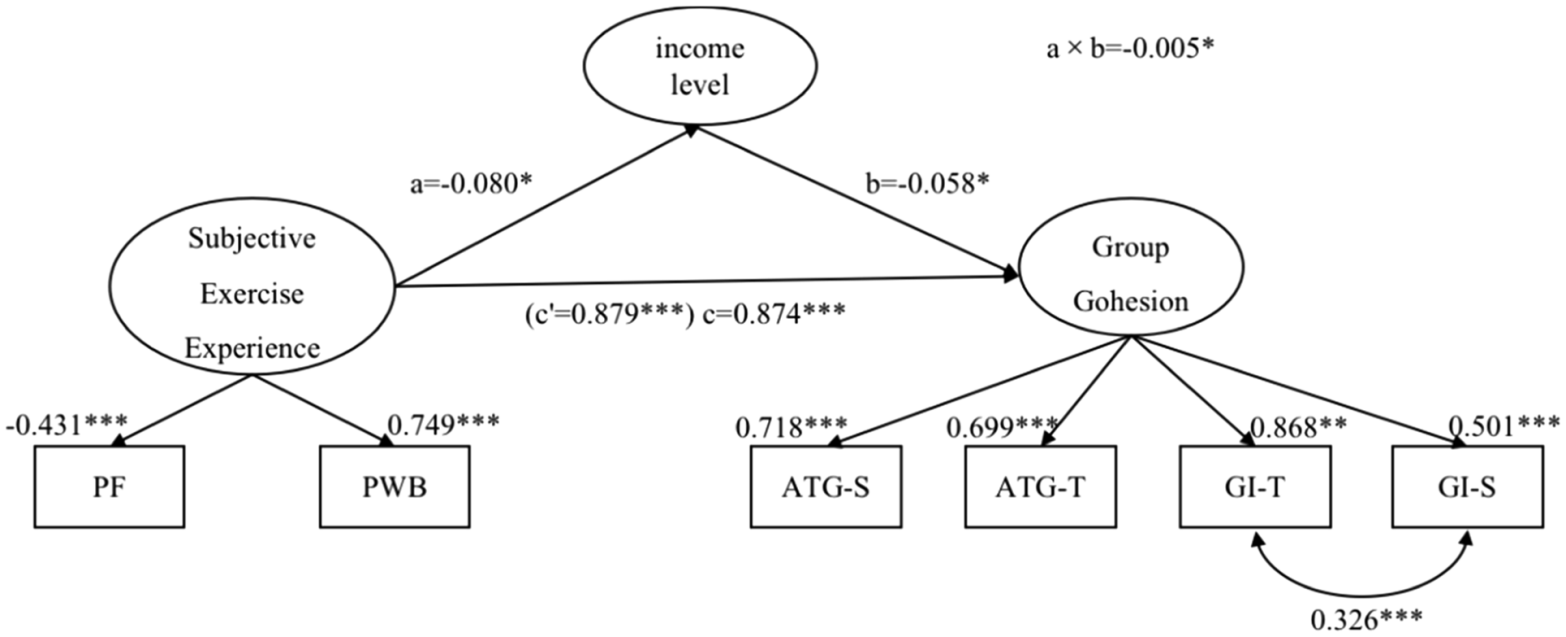
The mediating model of the subjective exercise experience, group cohesion, and income. PF, psychological fatigue; PWB, positive well-being; GI-T, group integration-task; GI-S, group integration-social; ATG-T, individual attractions to group-task; and ATG-S, individual attractions to group-social. Summary standardized path coefficients representing the effects of the subjective exercise experience and income rating on group cohesion. All values represent standardized estimates/coefficients. ^***^p<0.001; ^**^p<0.01; and ^*^p<0.05.

The mediation model of education did not fit the data (*χ*^2^/df=9.409, AGFI=0.935, CFI=0.958, GFI=0.972, RMSEA=0.085, SRMR=0.035, and TLI=0.926), and the indirect effect of the subjective exercise experience on group cohesion through education was not significant (*a*×*b*=−0.001, SE=0.002, 95% CI [−0.006, 0.001], *p*=0.176). The mediation model of income had an excellent fit to the data (*χ*^2^/df=4.901, AGFI=0.967, CFI=0.98, GFI=0.987, RMSEA=0.06, SRMR=0.04, and TLI=0.97). The detailed results of the mediation analysis are displayed in Figure 1, where the effect of the subjective exercise experience on the income level is illustrated as *a*; the effect of the income level on group cohesion is illustrated as *b*; and the indirect effect is the product of paths *a* and *b* and is illustrated as *a*×*b*. The direct effect and the total effect are illustrated as *c*’ and *c*, respectively.

Through the bootstrapping procedure, the results indicate that the indirect effect of the subjective exercise experience on group cohesion through income level was also significant (*a*×*b*=−0.005, SE=0.003, 95% CI [−0.003, 0.000], *p*=0.037). Approximately 77.6% of the variance in group cohesion was accounted for by the subjective exercise experience and income level. The direct effect of the subjective exercise experience on group cohesion was significant after controlling for the income level (*c*=0.874, SE=0.039, 95% CI [−0.951, −0.800], *p*=0.001). Additionally, the total effect was significant (*c*’=0.879, SE=0.038, 95% CI [−0.955, −0.806], *p*<0.001).

The evaluation of the specific paths from the subjective exercise experience to the income level and group cohesion indicated that the subjective exercise experience was a significantly positive predictor of group cohesion (*B*=−1.067, *β*=−0.874, SE=0.094, *p*=0.001) and a negative predictor of the income level (*B*=0.055, *β*=0.080, SE=0.026, *p*=0.035), which was a negative predictor of group cohesion (*B*=−0.105, *β*=−0.058, SE=0.052, *p*=0.044). This study also explored whether the duration of exercise had a mediating effect on the relationships between the subjective exercise experience and group cohesion. In contrast to expectation, this study did not find any significant association. In summary, the results demonstrate the presence of significant direct and indirect effects in the mediation model. The subjective exercise experience remained a significant predictor of group cohesion after including income level as a mediator, suggesting that the model involves partial mediation.

### Subjective Exercise Experience and Group Cohesion

**Figure 2:**
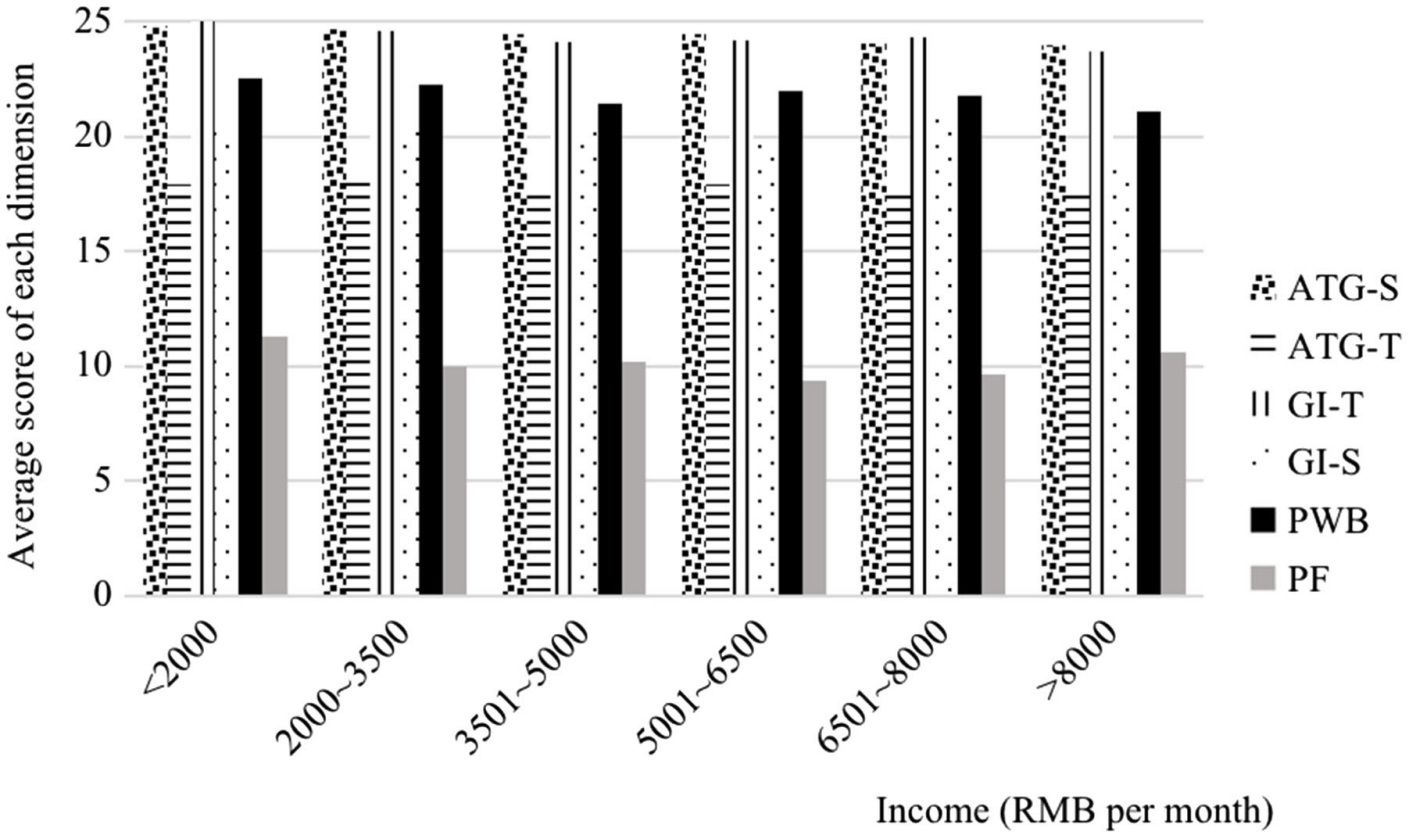
Differences in income level in the ratings of group cohesion and subjective exercise experience. PF, psychological fatigue; PWB, positive well-being; GI-T, group integration-task; GI-S, group integration-social; ATG-T, individual attractions to group-task; and ATG-S, individual attractions to group-social.

PWB, which represents the positive aspects of the subjective exercise experience, was strongly positively associated with group cohesion. In contrast, PF, which represents the negative aspects of the subjective exercise experience, was moderately negatively associated with group cohesion as shown in Figure 2.

### Income Level and Subjective Exercise Experience

There were several increases and decreases in the associations between the PWB and PF ratings and income. Negative and positive relations are related to the characteristics of regions (such as developing countries) and participants (such as retirees, Notice on Adjusting the Basic Pension for Retired Persons in 2020, 2020).

As displayed in Figure 2, the association between the income level and group cohesion was negative.

### Income Level and Social Aspect of Group Cohesion

The social aspect of group cohesion consisting of ATG-S and GI-S was defined by Carron et al. (1985) as a general orientation toward developing and maintaining social relationships within a group. According to Figure 2, an increasing income level had a greater effect on the reduction in GI-S than ATG-S, whereas the fifth income level (6,500–8,000 RMB) was the most strongly associated with GI-S.

### Income Level and Task Aspect of Group Cohesion

The task aspect of group cohesion consisting of ATG-T and GI-T was defined by Carron et al. (1985) as a general orientation toward achieving the group’s goals and objectives. Figure 2 shows that an increasing income level had a greater effect on the reduction in GI-T than ATG-T.

### Income Level and Individual Attractions to Group Cohesion

Individual attractions to the group represent the interaction among the motivations for an individual to remain in a group, i.e., the composite of the individual members’ feelings regarding the group, their personal role involvement, and involvement with other group members (Carron et al., 1985). Notably, increases in the income level had a minimal effect on ATG-T and ATG-S, except for the rating of ATG-T provided by the lower income-level group (<3,500 yuan), which increased with the income level.

### Income Level and Group Integration

Group integration represents the closeness, similarity, and bonding within the group as a whole, i.e., the degree of unification of the group (Carron et al., 1985). Figure 2 shows that an increasing income level had a greater effect on the reduction in GI-T than GI-S.

## Discussion

The current study found that first, square dance satisfied women’s physical and psychological needs partly, which is consistent with hypothesis 1. Second, PWB was positively associated with group cohesion, and in contrast, PF was negatively correlated with group cohesion, supporting hypothesis 2 of our study. However, a part of hypothesis 3 was supported because only income showed a mediating effect between the subjective exercise experience and group cohesion.

### Square Dance Satisfied Women’s Needs Partly

Recently, the physical and psychological problems of women have become major concerns worldwide (Biering, 2019). From a subjective view, square dance as a type of synchronizing dance group activity satisfied women’s physical and psychological health needs partly.

First, our study found that most women who perform square dance seek to satisfy physical health needs because they believe that square dance can not only help them maintain wellness but also enable them to build a good shape. Indeed, previous studies indicate that square dance, a music-related public group activity, promotes women’s physical health (Xiao and Hilton, 2019). More specifically, square dance is an entertaining way for people to improve their physical health, such as flexibility, lower extremity strength, and coordination (Yu et al., 2020). In addition, older women who dance synchronously in a group are more resistant to pain and have elevated pain thresholds (Tarr et al., 2016). Therefore, the physical health benefits of square dance could help women meet their physical health needs partly.

Second, notably, square dance also helps women satisfy psychological needs, such as increasing positive psychological moods and acquiring social opportunities. Given that women, especially middle-aged women, always feel lonely in their daily life (Lund et al., 2018), they are at an increased risk of developing negative mood disorders (Seedat et al., 2009). Therefore, women need to increase their positive effect and broaden their social circles to improve this condition. Indeed, previous studies indicate that physical group activities, such as square dance, effectively increase women’s positive effect (Seedat et al., 2009). In addition, our study found that most women choose to participate in square dance because they feel a sense of belonging to the group and gain more social interaction opportunities, such as communicating with others and meeting new friends. Participating in square dance meets women’s psychological needs (social demands) because synchronizing full-body dance activities help the participants increase their feelings of social closeness to each other in a group (Robertson et al., 2017). Specifically, when participants perform the same movements synchronously, there is coactivation of action and perception networks, which is believed to blur the sense of others and the self (Overy and Molnar-Szakacs, 2009), implying that participating in synchronous full-body dance contributes to a social bond among the participants in the group (Decety and Sommerville, 2003). Therefore, to meet their physical and psychological needs, women are more likely to participate in square dance.

### Subjective Exercise Experience and Group Cohesion

The current study showed that PWB was positively associated with group cohesion and that fatigue was negatively related to group cohesion, which is consistent with previous studies.

Previous studies indicate that group cohesion, such as social interaction, is positively associated with PWB (Lee and Ishii-Kuntz, 1987; Nezlek et al., 2002; Cramm and Nieboer, 2014, 2015), indicating that identification with a social group tends to promote PWB (Greenaway et al., 2015). However, anthropologists emphasize the important role of positive emotional states leading to social closeness in a synchronized dance group (Durkheim, 1915). Similarly, at least one recent study found that better psychological health also predicted higher group identifications (Miller et al., 2017), which supports the results of our study because group identification is the main reason why individuals are attracted to group activities (Brawley, 1979). These results are consistent with those reported by Benish-Weisman et al. (2015). In addition, notably, a panel analysis of nationally representative Australian data showed that group participation predicts well-being, while well-being is related to more group identifications (Ding et al., 2015), indicating that there is a reciprocal relationship between psychological well-being and group participation.

Second, fatigue is negatively related to group cohesion. When fatigue or negative effect is decreased by participating in square dance, group cohesion is increased. In fact, the relationship between fatigue and group cohesion has been less discussed in previous studies, but it is important to determine why square dance simultaneously helps decrease fatigue and promote group cohesion among women. When participating in square dance, people usually dance synchronously with different music accompaniments; listening to music appears to be rated as a very pleasant experience since it decreases PF (Guo et al., 2015), and social facilitation theory suggests that arousal levels increase in the presence of others (Zajonc and Sales, 1966) because emotional contagion is believed to occur through processes of unconscious motor mimicry, causing individuals to experience emotions parallel to those perceived by the group (Hatfield et al., 2011). However, notably, emotional experiences tend to be amplified in group settings through processes of social feedback (Garrido et al., 2017), suggesting that women’s fatigue is decreased by music and that group members also decrease PF by social connection in the group.

### The Mediating Effect of Income Level on the Relationship Between Subjective Exercise Experience and Group Cohesion

In addition, the current study tested whether the relationship between the subjective exercise experience and group cohesion is mediated by the income level, indicating that subjective exercise experiences are negatively related to income and that income is positively related to group cohesion. However, the mediating role of the income level in the relationship between subjective exercise experiences and group cohesion is less discussed in related studies.

Positive well-being is negatively associated with income. Currently, many researchers have different perspectives regarding the relationship between well-being and income. On the one hand, income growth results in an increase in well-being. On the other hand, given the happiness–income paradox described by Easterlin et al. (2010), over time, a higher rate of income growth does not result in a greater increase in happiness. Indeed, Miñarro et al. (2021) found that higher levels of subjective well-being can be achieved with a low income level, further challenging the perception that economic growth raises life satisfaction among low-income populations. Similarly, Shapiro and Taylor (2002) found that higher subjective well-being can also be achieved by low-income elderly individuals. For example, Howell and Howell (2008) examined the relationship between economic status and subjective well-being in different income groups in developing countries, and the results showed that this relationship was the strongest in the sample of low-income developing economies but the weakest in the sample of high-income developing economies. This phenomenon is most likely due to the happy waterwheel theory, which posits that as the income level increases, the material desires of individuals continue to increase, which makes them gradually adapt to the reality of income increases and reduces the happiness that wealth brings (Knight, 2012; Tsutsui and Ohtake, 2016).

Furthermore, fatigue is positively related to income. Community-based studies employing bivariate analyses have shown a relatively consistent positive association between low income and negative effect (Lund et al., 2010). Similarly, over 12-month periods in India (Patel et al., 2006) and Taiwan (Seplaki et al., 2006), positive associations between low income and negative emotion have been reported. In addition, Wayne (2008) found that African-Americans in high-middle classes reported more fatigue than African-Americans in lower classes, indicating that people may have more fatigue when they are in a high income level, which is an important factor predicting social class (Featherman et al., 1975).

In addition, income is negatively associated with group cohesion, indicating that low income may promote group cohesion. For example, communities of older adults with a relatively low socioeconomic status, such as low-income attributes, gather (Lai et al., 2021), indicating that low income may predict high cohesion and that participation is considerably linked to belonging. Chinese people are more collectivistic than Westerners (Oyserman et al., 2002) because social capital in China resides largely in families and other narrow circles of social relationships, implying that people may participate in social activities involving their circles and trust those who belong to the same in-group (Allik and Realo, 2004).

Ultimately, the study also explored whether the duration of square dance has a mediating effect on the relationship between the subjective exercise experience and group cohesion, but our study did not find any significant association likely because the duration of square dance is not related to group cohesion, and Steca et al. (2013) also found similar results showing that there is no significant association between group cohesion and the duration of exercise in basketball and soccer teams. Therefore, these results support our initial prediction that the relationship between the subjective exercise experience and group cohesion is mediated by the income level.

Nevertheless, this study has several limitations. Both income and education were significantly correlated with the subjective exercise experience and group cohesion, but the mediating effect of education was not proven by the results. Therefore, the relations among these four variables require further investigations in the future. Second, studies should increase the sample size of participants from different cultures and countries and investigate the correlation between income and the motivation to participate in square dance (Kurková and Maertin, 2014). Furthermore, this study involved a random sampling survey, and 0.27% of the participants in our study were postgraduates; future studies should consider male participants and various age groups and collect more comprehensive and specific geographical information.

Moreover, future studies should conduct a longitudinal analysis to identify changes in the subjective exercise experience, group cohesion, and other geographical information and the duration of such associations. Despite these limitations, this study revealed a mediator of the relationship between the subjective exercise experience and group cohesion, i.e., the income level of the participants. Given the strong association between a positive exercise experience and group cohesion through income level, understanding this mechanism underlying group exercising behavior is an important contribution.

## Conclusion

In conclusion, our research found that women’s motivation to participate in square dance and square dance satisfied women’s physical and psychological needs partly. Second, PWB was positively associated with group cohesion, and fatigue was negatively related to group cohesion. Third, our research found evidence that the subjective exercise experience remained a significant predictor of group cohesion after including income level as a mediator, suggesting that the model was a partial mediation. Compared to previous studies, our findings explore a wide range of participants in China and use the Subjective Exercise Experiences Scale and Group Environment Questionnaire to study a new physical activity of national fitness, which may provide guidance for sports psychologists and square dance teams.

In addition, since early 2020, due to the breakout of the new coronavirus epidemic (COVID-19), the potential burden on the psychological health of the general public has increased (Burhamah et al., 2020). Notably, physical exercise benefits individuals by increasing positive feeling states and decreasing negative emotions (Bartholomew and Miller, 2002). Given that square dance can not only promote people’s physical and psychological health but also has no restrictions in space and time, individuals can practice square dance at home during the COVID-19 pandemic (Cheng and Li, 2020; Su, 2020). For example, the news has reported that doctors and patients participate in square dance in mobile cabin hospitals in China, which has a good impact on their psychological mood. In addition, people can share videos of their dancing and communicate with their friends *via* WeChat or TikTok (New Highlights in the Promotion of Home Scientific Fitness Methods, 2020). Therefore, the effects of square dance deserve more empirical and applied research. We believe that square dance activities may encourage people to perform exercises worldwide.

## Data Availability Statement

The original contributions presented in the study are included in the article/supplementary material, further inquiries can be directed to the corresponding authors.

## Ethics Statement

The studies involving human participants were reviewed and approved by Beijing Sport University Institutional Review Board (BSU IRB), (Ethical Approval Document No. 2020156H). The patients/participants provided their written informed consent to participate in this study. Written informed consent was obtained from the individual(s) for the publication of any potentially identifiable images or data included in this article.

## Author Contributions

All authors listed have made a substantial, direct and intellectual contribution to the work, and approved it for publication.

## Funding

This work was supported by the Fundamental Research Funds for the Central Universities (No. BSU2020030) to HF.

## Conflict of Interest

The authors declare that the research was conducted in the absence of any commercial or financial relationships that could be construed as a potential conflict of interest.

## Publisher’s Note

All claims expressed in this article are solely those of the authors and do not necessarily represent those of their affiliated organizations, or those of the publisher, the editors and the reviewers. Any product that may be evaluated in this article, or claim that may be made by its manufacturer, is not guaranteed or endorsed by the publisher.
